# Two Curious Bullet Wounds of the Neck

**Published:** 1915-07-17

**Authors:** 


### TWO CURIOUS BULLET WOUNDS OF THE NECK.

### By a Medical Officer at a Base in France.

Among the wounded admitted to base hospitals the percentage of wounds of the neck is always a low one; partly because the neck is a narrow target, partly because many wounds in that part are immediately or fairly speedily mortal. Pieces of shell and shrapnel are found embedded in the neck as often in proportion, perhaps, as in other wounded parts; but rifle bullets do not often present themselves for extraction. This is, no doubt, due to the fact that the soft tissues of the neck do not offer sufficient resistance to the flight of a bullet to arrest its course, unless the latter's velocity is already three parts spent, as by passing through the parapet of a trench or other obstacle; while those projectiles which are arrested by the spinal column are likely to cause death before the patient reaches the base.

Within the last few weeks, however, I have had occasion to extract four rifle bullets as well as several pieces of shell from the neck in soldiers admitted to a general hospital, and two of these cases were distinctly unusual. In the first case a soldier was hit by a stray bullet while employed more than a mile behind the trenches. The bullet entered on the right side of the neck just outside the sternomastoid muscle at about the level of the cricoid cartilage. The wound of entrance was a clean longitudinal slit, so small that it was thought to be due to a splinter of shell. On admission to hospital there was surgical emphysema of the right side of the neck, extending over the clavicle on to the upper part of the chest. The patient was comfortable, but looked ill; his temperature was 100° F. A penetrating wound of the apex of the right lung was suspected, and the man was kept in bed. After two or three days his temperature fell to normal, and the surgical emphysema disappeared. About this time he began to complain of weakness in the left hand. Investigation showed that there was no complete paralysis of any muscle, but there was certainly some weakness of the grip, and it was thought that the deltoid was also a trifle flattened. A screen examination showed a rifle bullet on the left side of the neck, and palpation then revealed it lying deep to the left sternomastoid with the point towards the skull. The base was towards the mediastinum and about a quarter of an inch above the level of the inner end of the clavicle.

Next day an operation was performed for the removal of the bullet. An incision was made parallel to the sternomastoid, along the inner border of that muscle. Careful dissection was necessary in exposing the carotid sheath and its component parts, as there was a certain small amount of inflammatory matting of the tissues. After defining and exposing the internal jugular vein, I retracted it aside and pushed down a blunt dissector between the vagus nerve and the carotid artery. On separating these two structures the bullet was found lying behind them, resting against the spine; it was extracted without difficulty. The wound was carefully closed with a small drainage tube in its lower corner; and except for a few drops of sero-pus from one small point it healed by first intention. In a short time the patient was discharged to a hospital ship with his wounds soundly healed and with complete recovery of the function of the left arm.

There can be little doubt that in this case a nearly spent bullet entered the neck, traversed the trachea from right to left, and came to rest between the carotid artery and the spine in a situation where it pressed slightly upon the last cervical nerve-root; but how it got into such a position without injuring the important and delicate vessels and nerves by which it was surrounded remains a mystery, especially as it is clear that the bullet made at least one half-turn on a transverse axis after it entered the skin, probably after it passed through the trachea.

The second soldier had an even narrower escape of his life. He was admitted with a minute slit-like wound on the back of the neck, rather to the right of the midline; this wound was quite clean and never gave any trouble. He complained of difficulty in swallowing, or rather of discomfort on attempting the act. Palpation revealed a bullet under the right sternomastoid at the level of the hyoid bone. This bullet seemed not so deep as that in the case already narrated had seemed, though it was evidently deep to the sternomastoid. An incision over this muscle, and parallel to its fibres, was made, and the point of the bullet could then be felt directed forwards and embedded in the muscle itself. The muscle was carefully incised just enough to permit of the bullet being seized in forceps and pulled out, point first. No sooner had this manœuvre been completed than a furious gush of venous blood took place from the depths of the wound. This was at once controlled by sponge pressure, and the rent in the muscle was enlarged in order to get at the source of the bleeding. After some painstaking dissection, interrupted by spurts of blood whenever pressure was relaxed, a curious condition was revealed. At the bottom of the bed in which the bullet had been lying was an oval fenestra in the carotid sheath on its outer aspect; this was not an operation artefact, but had been caused by the passage of the bullet from behind forwards. Inside this aperture was a notch or bite, as it were, in the outer side of the internal jugular vein. With some difficulty this was picked up in a Spencer Wells' clip and a lateral ligature applied to the vessel. All bleeding immediately ceased, and the wound was sewn up in three layers without drainage; it healed speedily by first intention throughout.

What had happened in this case was that the bullet, which must have been almost completely spent (probably by passing through the parapet of the trench in which the soldier was), cut a small hole in the outer side of the jugular; but coming to rest with its point in the sternomastoid and its base firmly held in the carotid sheath, the hole in the vessel was, as it were, plugged until the bullet was removed at the operation. There was no infiltration of blood in the tissues at all to warn one of injury to the vessels, nor was there any transmitted pulsation when the point of the bullet came into view during incision of the sternomastoid. Had a lateral ligature not turned out to be possible—it was exceedingly difficult—the only alternative would have been a dissection of the jugular, with ligature above and below the wound—not an easy operation in all the circumstances, and one I was quite glad to find unnecessary.

The only cases of important nerve injury in neck wounds which I have seen have been due to shells. In two cases there has been extensive, though not complete, paralysis of the arm on the affected side. In one of these cases there was surgical emphysema as a complication, due to injury of the apex of the lung; and in another the patient was suffering, in addition to his wounds, from enteric fever, of which he had experienced the early symptoms some few days before he was wounded.

